# Qualitative systematic review of barriers and facilitators to self-management of chronic obstructive pulmonary disease: views of patients and healthcare professionals

**DOI:** 10.1038/s41533-017-0069-z

**Published:** 2018-01-17

**Authors:** Siân Russell, Oladapo J. Ogunbayo, James J. Newham, Karen Heslop-Marshall, Paul Netts, Barbara Hanratty, Fiona Beyer, Eileen Kaner

**Affiliations:** 1 Institute of Health and Society, Newcastle University, Baddiley-Clark Building, Richardson Road, Newcastle upon Tyne, NE2 4AX USA; 2 Department of Primary Care & Public Health Sciences, King’s College London, London, UK; 3 NHS Newcastle Gateshead Clinical Commissioning Group, Newcastle upon Tyne, UK

## Abstract

Self-management interventions for chronic obstructive pulmonary disease (COPD) can improve quality of life, reduce hospital admissions, and improve symptoms. However, many factors impede engagement for patients and practitioners. Qualitative research, with its focus on subjective experience, can provide invaluable insights into such factors. Therefore, a systematic review and synthesis of qualitative evidence on COPD self-management from the perspective of patients, carers, and practitioners was conducted. Following a systematic search and screening, 31 studies were appraised and data extracted for analysis. This review found that patients can adapt to COPD; however, learning to self-manage is often a protracted process. Emotional needs are considerable; frustration, depression, and anxiety are common. In addition, patients can face an assortment of losses and limitations on their lifestyle and social interaction. Over time, COPD can consume their existence, reducing motivation. Support from family can prove vital, yet tinged with ambivalence and burden. Practitioners may not have sufficient time, resources, or appropriate skills or confidence to provide effective self-management support, particularly in regard to patients’ psychosocial needs. This can compound patients’ capability to engage in self-management. For COPD self-management to be effective, patients’ psychosocial needs must be prioritised alongside medication and exacerbation management. In addition, patients’ personal beliefs regarding COPD and its management should be reviewed periodically to avoid problematic behaviours and enhance positive adaptions to the disease. Patients with COPD are not a homogenous group and no one intervention will prove effective for all. Finally, practitioners require greater education, training, and support to successfully assist patients.

## Introduction

Chronic obstructive pulmonary disease (COPD) is typically characterised by breathlessness (dyspnoea), chronic airway obstruction, and chronic cough with sputum production. COPD, caused predominately by smoking, is a leading cause of mortality and disability worldwide and represents a socioeconomic burden for developed and developing countries.^1^ Within the UK, COPD is the second largest cause of unplanned hospital admissions^2,3^ and costs the National Health Service approximately £810–£930 m annually.^3^

COPD is incurable. Patients face both acute symptom exacerbations and gradual decline in lung function over time, negatively impacting upon activities of daily living, resulting in depression and anxiety, and reducing health quality of life (HRQoL).^4–7^ Such problems can be compounded by comorbidities (e.g., diabetes, osteoporosis, hypertension, lung cancer).^8–10^ Disease incidence and poor outcomes are associated with socioeconomic deprivation, lower educational attainment, childhood disadvantage, and marginalised communities.^11–14^ As such, COPD can be viewed as socially patterned, and associated with inequality.

It has become accepted that people with chronic conditions should be actively engaged in the self-management of their condition(s). Self-management relates to “an individual’s ability to detect and manage symptoms, treatment, physical and psychosocial consequences, and lifestyle changes inherent in living with a chronic condition”.^15^ Practitioners can facilitate self-management through strategies of support (e.g., patient education, goal setting).^16^ The “ultimate goals” of COPD self-management, according to Effing et al., are improving and maintaining physical health, lessening the impact of symptoms and impairments, increasing emotional, social, and psychological well-being while creating “effective alliances” with family, practitioners, and community.^8^ Given the broad spectrum of issues that fall under the umbrella of self-management, interventions are wide ranging and heterogeneous in focus and delivery.^17–21^

Recent reviews focusing on the effectiveness of COPD self-management interventions suggest interventions can improve HRQoL and disease symptoms, reducing hospital admissions.^17,19,20^ However, patient engagement can be influenced by structural, disease-related, social, and psychological factors (e.g., short consultations, comorbidities, health literacy, social support, depression, anxiety).^22–26^ The social, emotional, and medical needs of those with COPD are varied and complex, making it a challenging condition to self-manage and support. In their recent and extensive review of COPD self-management interventions, Jordan et al. called for further qualitative work to explore barriers and facilitators to COPD self-management.^19^ Qualitative research, with its focus on subjective experience, is well placed to enhance understanding of such factors,^27^ and enrich guidelines to support improved practice.^28^ This systematic review of qualitative research aims to provide an in-depth insight into the barriers and facilitators to self-management from the perspectives of COPD patients and practitioners involved in the care of COPD patients. It forms part of a wider programme of research exploring COPD self-management from which a meta-analysis and an original qualitative research paper have been published.^20,22^

## Results

**Fig. 1:**
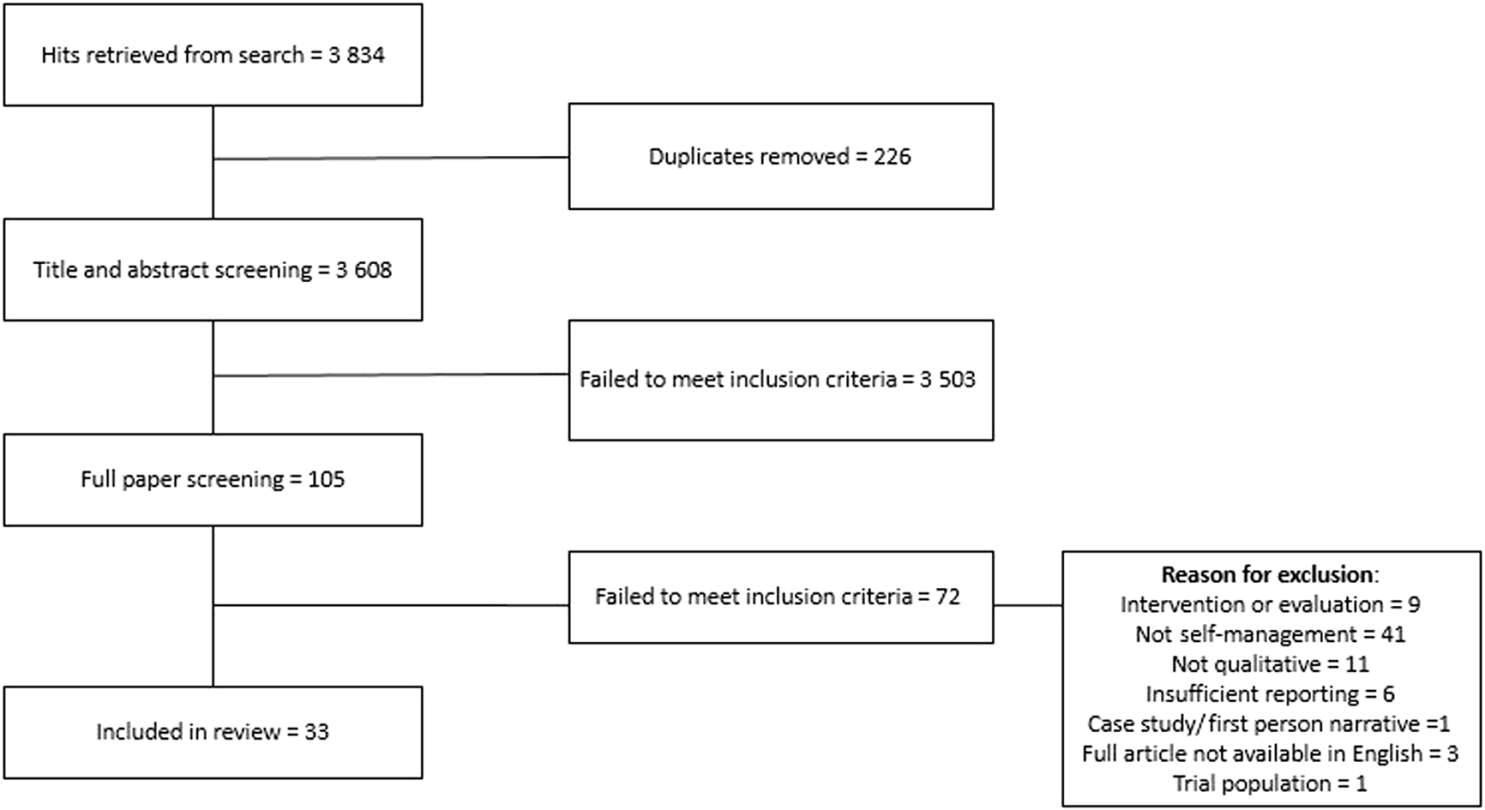
PRISMA diagram of papers eligible for data extraction

The search retrieved 3608 unique articles. Following title and abstract screening 105 were screened on full text, resulting in the exclusion of 72 articles. Of the remaining articles, two by Andersen et al.^29,30^ concerned the same study and two by Chen et al. also reported on one study.^31,32^ In both instances these were analysed as one. The paper by Ogunbayo et al. is from the same research team as this review.^22^ Thus, this review reports on 31 studies. Figure 1 provides a PRISMA diagram of papers eligible for inclusion.

### Quality

The overall quality of the included papers was generally “very good” or “good” (see Table 1) indicating a relatively high-quality body of work.

**Table 1:** Quality of included papers

Author	Aims scope	Ethical dimension	Study design	Rigour	Analysis procedure	Depth/detail	Credibility	Relevance transferability	Contribution to knowledge	Overall quality
Andersen et al. (a) and (b)	VG	VG	VG	G	VG	G	VG	VG	VG	**VG**
Apps et al.	VG	VG	VG	VG	VG	VG	G	VG	VG	VG
Boeckxstaens et al.	VG	VG	VG	VG	VG	G	VG	VG	VG	**VG**
Brandt	G	VG	VG	VG	VG	L	L	G	G	***G***
Brien et al.	VG	VG	G	G	G	G	G	G	G	**G**
Chang et al.	VG	G	G	G	VG	G	G	G	G	**G**
Chen et al. (a) and (b)	VG	VG	G	VG	VG	VG	L	VG	VG	**VG**
Cicutto et al.	VG	VG	VG	VG	VG	VG	VG	VG	VG	**VG**
Duangpaeng et al.	VG	G	G	G	G	G	L	G	G	***G***
Ehrlich et al.	VG	VG	VG	VG	VG	VG	G	VG	VG	**VG**
Fotokain et al.	G	VG	G	VG	VG	G	G	G	G	**G**
Gysels and Higginson	VG	VG	VG	VG	VG	VG	VG	VG	VG	**VG**
Harris et al.	VG	G	VG	VG	VG	VG	VG	VG	VG	**VG**
Harrison et al.	VG	VG	VG	VG	VG	VG	VG	VG	VG	**VG**
Hillebregt et al.	G	VG	VG	VG	VG	G	G	VG	VG	**VG**
Hyde et al.	VG	G	VG	VG	VG	VG	G	VG	VG	**VG**
Johnston et al. (a)	VG	VG	VG	VG	VG	G	G	VG	VG	**VG**
Johnston et al. (b)	VG	G	VG	VG	G	VG	VG	VG	VG	**VG**
Kayyali et al.	VG	G	G	G	G	G	L	G	G	**G**
McCabe et al.	VG	G	VG	G	G	G	G	VG	VG	***G***
Ogunbayo et al.	VG	VG	G	VG	VG	G	VG	VG	VG	**VG**
Panos et al.	VG	G	VG	VG	VG	VG	G	VG	VG	**VG**
Robinson et al.	VG	VG	G	VG	VG	G	VG	VG	VG	**VG**
Sheridan et al.	VG	VG	VG	G	L	VG	VG	VG	VG	**VG**
Stellefson et al.	VG	VG	VG	VG	VG	VG	VG	G	G	**VG**
Summers et al.	VG	VG	G	VG	VG	G	VG	VG	VG	**VG**
Verbrugge et al.	VG	G	VG	VG	VG	VG	VG	VG	VG	**VG**
Wong et al.	VG	VG	VG	G	G	VG	G	VG	VG	**VG**
Wortz et al.	VG	VG	G	G	G	VG	VG	VG	VG	**VG**
Young et al.	VG	VG	VG	G	G	VG	G	VG	VG	**VG**
Zakrisson and Hagglund	VG	G	G	G	G	VG	G	VG	VG	***G***

*L *low, *G* good, *VG* very good

### Overview of included papers

Included studies were published between 2002 and 2017. Eighteen papers came from Western countries (European countries, North America, Australia, and New Zealand), with only five articles from elsewhere (Iran, Taiwan, Thailand, and Malaysia). Ten papers explicitly referred to self-management within their research aims. The remaining papers referred to self-management elsewhere in the narrative. The majority were interview studies. Thematic analysis, grounded theory, and constant comparison were the most common approaches to data analysis.

Seventeen papers focused on COPD patients,^31–47^ eight on practitioners,^22,48–54^ three on patients and practitioners,^55–57^ three on patients and carers,^29,30,58,59^ and one included all three categories.^60^ The combined population sample included 499 people with COPD, 143 practitioners, and 36 carers.

The mean age of the COPD patients was 69, calculated from 16 papers where age was specifically reported. Around two-thirds (62%) of participants were male, with three studies containing male-only samples.^31,42,55^ Sixteen papers failed to report ethnicity. From the remaining papers, 50% were Caucasian. Twelve papers did not report comorbidity. Of the five remaining papers the mean number of comorbidities was 4 (min = 1/max = 10) (see Table 2).

**Table 2:** Patient characteristics

Author^ref.^	Country	Sample size	Mean age (years)	Gender	Ethnicity	No. of co-morbidities/co-existing conditions
Andersen et al.^29, 30^	Denmark	15	69	Female: 10	NR	NR
				Male: 5		
Apps et al.^33^	UK	15	69	Female: 9	NR	NR
				Male: 6		
Brandt^35^	USA	28	69	Female: 9	Caucasian: 27	NR
				Male: 19	Black/African American: 1	
Brien et al.^46^	UK	34	72	Female: 13	NR	Mean: 3.4
				Male: 21		Min: NR
						Max: NR
Boeckxstaens et al.^34^	Belgium	7	NR	Female: 3	NR	Mean: 6
				Male: 4		Min: 2
						Max: 9
Chang et al.^47^	Taiwan	14	71	Female: 2	NR	NR
				Male: 12		
Chen et al.^31, 32^	Taiwan	19	74	Female: 0	NR	NR
				Male: 19		
Cicutto et al.^41^	Canada	42	71	Female: 19	NR	NR
				Male: 23		
Duangpaeng et al.^36^	Thailand	31	NR	NR	Thai: 31	NR
Ehrlich et al.^37^	Australia	9	69	Female: 5	NR	NR
				Male: 4		
Fotokain et al.^60^	Iran	15	NR	NR	NR	NR
Gysels and Higginson^58^	UK	18	NR	Female: 11	NR	NR
				Male: 7		
Harris et al.^38^	UK	16	67	Female: 4	NR	NR
				Male: 12		
Harrison et al.^39^	UK	6	75	Female: 4	NR	Mean: 5.5
				Male: 2		Min: 1
						Max: 8
Hillebregt et al.^57^	The Netherlands	17	NR	NR	NR	NR
Hyde et al.^56^	Ireland	15	NR	Female: 8	NR	NR
				Male: 7		
McCabe et al.^40^	Ireland	32	67	Female: 17	NR	Reported in percentages by co-morbidities/co-existing conditions
				Male: 15		
Panos et al.^42^	USA	42	65	Female: 0	Caucasian: 30	NR
				Male: 42	African American: 11	
					NR: 1	
Robinson et al.^59^	Australia	18	NR	Female: 6	NR	NR
				Male: 12		
Sheridan et al.^43^	New Zealand	29	72	Female: 14	European: 10	Mean: 3
				Male: 15	Pacific Island: 19	Min: 1
						Max: 8
Stellefson et al.^44^	USA	12	67	Female: 8	Caucasian: 7	NR
				Male: 4	African American: 5	
Wong et al.^55^	Malaysia	18	72	Female: 0	Chinese: 8	NR
				Male: 18	Malay: 7	
					Indian: 3	
Wortz et al.^45^	USA	47	68	Female: 22	Caucasian: 41	Mean: 2
				Male: 25	Black: 5	Min: NR
					Other: 1	Max: NR
	Total	499	70	Female: 164	Caucasian: 105	Reported: 5 papers
				Male: 272	Asian: 49	Average mean: 4
				NR: 63	Black/African American: 22	Average min: 1
					Pacific Island: 19	Average max: 8
					“European”: 10	NR: 12 papers
					Other: 1	
					NR: 293	

Practitioner participants included 58 respiratory specialists, 42 GPs, 18 nurses (non-respiratory), 11 allied health professionals, 2 pharmacists, and 25 other professionals (registrars, interns, community matron, and an exercise instructor). Of the seven papers where gender and ethnicity was reported, 82% of participants were female and 68% were Caucasian (see Table 3).

**Table 3:** Practitioner characteristics

Author^ref.^	Country	Sample size	Occupation	COPD expertise/engagement	Gender	Ethnicity	Mean age (years)
Fotokain et al.^60^	Iran	5	Nurses = 3	NR	NR	NR	NR
			Physiotherapist = 1				
			Pulmonologist = 1				
Hillebregt et al.^57^	The Netherlands	10	NR	NR	NR	NR	NR
Hyde et al.^56^	Ireland	5	Practice nurse = 3	Delivered usual care to patient participants with COPD	Female: 3	NR	NR
			General practitioner = 2	Mean years in post: 13	Male: 2		
Johnston et al.^49^	Australia	16	Hospital-based medical practitioners = 9	“Actively involved” in the care of COPD patients in primary and tertiary care settings	NR	NR	NR
			General practitioner = 7				
Johnston et al.^48^	Australia	12	General practitioner = 12	“Actively involved” in the care of COPD patients in a tertiary care setting	Female: 10Male: 2	NR	NR
Kayyali et al.^54^	UK	23	General practitioner = 1	NR	NR	NR	NR
			Specialist doctors = 13				
			Nurses = 6				
			Physiotherapists = 3				
Ogunbayo et al.^22^	UK	20	General practitioner = 2	Multidisciplinary healthcare teams involved in COPD care	Female: 15 Male: 5	Caucasian: 20	45
			*Nurses*				
			Practice nurse = 2				
			Respiratory nurse = 1				
			*Pharmacy*				
			Practice pharmacist = 1				
			Community pharmacist = 1				
			*Respiratory*				
			Specialist respiratory/COPD practitioners = 6				
			Consultant respiratory physician = 1				
			*Allied health professionals*				
			Physiologist = 1				
			Physiotherapist = 1				
			Occupational therapist = 1				
			*Other*				
			Community matron = 2				
			Exercise instructor = 1				
Summers et al.^53^	UK	17	Respiratory physiotherapists = 17	With ⩾12 months current or previous experience of working with patients with COPD in a non-acute setting	Female: 13Male: 4	Caucasian: 14Black African: 1White S African: 2	NR
Verbrugge et al.^50^	The Netherlands	14	Respiratory nurses = 14	Nurse-led clinics with a population of COPD patients in general hospitals, homecare organisations, and a university hospital	Female: 14Male: 0	NR	39
				Mean years of experience: 5			
Wong et al.^55^	Malaysia	18	General practitioner = 18	Manage COPD patients within a hospital chest clinic or primary care	Female: 13Male: 5	Malay: 7Indian: 5Chinese: 4Other Asian: 2	NR
Young et al.^52^	UK	14	*Allied health professional*Physiotherapists = 3Occupational therapist = 1	Currently or recently (last 12 months) working with COPD patientsPrimary, community, and secondary care	Female: 14Male: 0	Caucasian: 13African Caribbean: 1	NR
			*Nurse *(*Respiratory*)				
			Respiratory research nurse = 3				
			Community respiratory nurses = 2				
			Respiratory nurse = 1				
			*Nurse *(*other*)				
			Practice nurse = 3				
			Nurse practitioner = 1				
Zakrisson and Hagglund^51^	Sweden	12	Asthma/COPD nurses = 12	Specialist / University education in asthma/COPD: 8No specialist education: 2	NR	NR	NR
				Median years of experience in asthma/COPD clinics: 7			
				Primary care setting			
	Total	166	Respiratory = 58		Female: 82	Caucasian: 47	NA
			General practitioner = 42		Male: 18	Asian: 18	
			Nurse = 18Other practitioner = 25Allied health professional = 11Pharmacists = 2NR = 10		NR: 43	White S African: 2 African Caribbean: 1Black African: 1NR: 74	

Data concerning carers was limited. From the papers where gender and relation to participant were reported, the majority were spouses (78%) and were female (67%) (see Table 4).

**Table 4:** Carer/family member characteristics

Author^ref.^	Country	Sample size	Relation to patient	Gender
Andersen et al.^29^	Denmark	12	Spouse: 8	Female: 9
			Daughter: 4	Male: 3
Gysels and Higginson^58^	UK	11	Spouse: 10	NR
			Daughter: 1	
Fotokain et al.^60^	Iran	4	NR	NR
Robinson et al.^59^	Australia	9	NR	Female: 5
				Male: 4
	Total	36	Spouse: 18	Female: 14
			Daughter: 5	Male: 7
			NR: 13	NR: 15

### Self-management definitions

Definitions and explanations of self-management across the papers varied. As detailed in Table 5, 10 papers offered no clear definition or explanation, 8 referred to emotional or psychological elements, and 7 highlighted well-being or quality of life. Nineteen papers characterised self-management, at least in part, in terms of tasks, skills, and techniques, self-regulation or self-monitoring. Eleven papers referred to disease knowledge.

**Table 5:** Definitions of self-management

Author	Definition or explanation	Emotional or psychological	Well-being or quality of life	Skills, tasks monitoring, self-regulation	Disease knowledge
Andersen et al. (a) and (b)	x				
Apps et al.	√		√	√	
Brandt	√			√	√
Brien et al.	x				
Boeckxstaens et al.	√			√	√
Chang et al.	x				
Chen et al. (a) and (b)	√			√	√
Cicutto et al.	√	√		√	
Duangpaeng et al.	√			√	√
Ehrlich et al.	√			√	
Hillebregt et al.	√	√		√	
Fotokain et al.	√		√		
Gysels and Higginson	√			√	√
Harris et al.	√	√		√	
Harrison et al.	x				
Hyde et al.	√				√
Johnston et al. (a)	√	√		√	
Johnston et al. (b)	x				
Kayyali et al.	x				
McCabe et al.	√		√	√	√
Ogunbayo et al.	√	√		√	
Panos et al.	x				
Robinson et al.	x				
Sheridan et al.	√	√		√	√
Stellefson et al.	√	√		√	√
Summers et al.	x				
Verbrugge et al.	√	√	√	√	
Wong et al.	√		√	√	√
Wortz et al.	√		√	√	√
Young et al.	√		√	√	
Zakrisson and Hagglund	x				

### Findings

Below the findings that emerged from the analysis are presented and discussed. For participant quotes the following key applies: P = Patient, HP = Healthcare practitioner, C = Carer.

### Knowledge, understanding, beliefs, and communication

Patient knowledge and understanding of COPD appeared to be interwoven with the individual’s “lifeworld” (subjective sense of self and the external world, shaped by personal experiences). As Ehrlich reported:

… self-generated intrinsic information and externally available information was processed through an interpretive filter aimed at determining the relevance and plausibility of that information in participants’ own lives.^37^

The lifeworld interpretation could be positive, as patients adapted to the condition; however, it also enabled patients to rationalise problematic behaviours such as continuing to smoke or ignoring advice of practitioners. For example, Apps et al. found patients’ beliefs about medication could impact upon their adherence to prescribed medications.

I’m not using my inhalers so much … I might be wrong on this, but I’m thinking if I don’t have to use the inhalers too much now, if I get worse, I’ve still got the inhalers to use before I have to go on the dreaded oxygen. (P)^33^

In addition, patient knowledge of COPD was reported as limited within patient focused papers and by practitioners. Patients failed to understand terminology, conflating COPD with asthma, did not understanding the progressive/incurable nature of COPD, and were confused regarding exercises and how to recognise and respond to exacerbations.^33,34,42–45,55,56^ Family/carers could often fill this gap by taking responsibility for asking questions and implementing information.^29,59^ Lack of understanding or confusion could lead to frustration and have implications for patient’s confidence in undertaking self-management activities.^33,38,45^ However, eight studies reported that patients had either received limited or no information from practitioners.^33,38,43–45,55,56,58^ Patients felt frustration due to conflicting information received from different practitioners and external sources, a lack of opportunity to ask questions within consultations, and medicine being prioritised over lifestyle concerns. Conversely, practitioners were concerned about patients’ confidence, literacy, health literacy, and recall.^49,54^ Practitioners could make assumptions regarding patients ability to understand COPD, resulting in misleading terms such as asthma or “breathing problems” being used.^55,56^

… Should I give him a leaflet? Is that enough? Can the patient read? …How long does he remember it …? (HP)^54^

A lot of them, they don’t even know what is COPD. If symptoms are mainly breathlessness, then it’s ‘asthma’ (HP)^55^

Practitioners understanding of self-management often seemed narrow, focusing on exacerbation and medication, with practitioners being unfamiliar with goal setting and deficient at promoting physical activity, breathing exercises, and good diet.^52,56^ Practitioners’ often felt they lacked appropriate knowledge, skills, or education to support self-management, and instead advice focused on adherence to specific behaviours such as smoking cessation and medication.^49,56^ Zakrisson and Hagglund reported that nurses felt a sense an “insufficiency” of skills in health education:

But that [motivational interviewing] is a technique I still haven’t really mastered. […] I find it difficult to steer the interview and simultaneously really reflect what has been said while you still want to get your message across. (HP)^51^

Young et al. reported that many nurses and allied health professionals viewed self-management as outside their daily practice, choosing to refer patients to others for support, highlighting the need for an increased role of pharmacy in self-management.^52,54^ Johnston et al. reported a lack of role clarity regarding pulmonary rehabilitation referrals.^48^ Behaviour change and patient education were viewed as complex, time consuming, and difficult for patients to adopt.^48–52,54^ In addition, communication between different practitioners could be problematic, complicating the care pathway:^22,54^

Communication with other professionals is a challenge. / Different people involved in the care. So it’s very complex … (HPs)^54^

### Patient–practitioner relationships

From the patient perspective, practitioners were reported to be important and necessary, and there were some positive interactions, particularly in reference to pulmonary rehabilitation^41^ which was deemed as beneficial for self-management and maintaining exercise.^31^ A multidisciplinary team appeared to be beneficial for providing relevant information (e.g., diet, exercise). However, productive relationships with practitioners could be impeded by: problems of inadequate information; a lack of opportunities to ask questions; feeling rushed by practitioners; a lack of faith in practitioners; lengthy waiting times; or advice that conflicted with patients’ perceptions.^39,45,55,59^ Patients could sometimes delay seeking professional support as they wished to avoid hospital or they felt services were too stretched to treat them.^39^ In addition, some did not want to “bother” their GP,^35^ while others believed they could “tough it out”^42^ or were worried about being judged due to previous or continued smoking.^39,56^ In addition, patients differed across the studies in terms of level of dependence they should have on practitioners or conversely the level of personal responsibility they believe they should take for disease management:

We cannot depend on ourselves. We need someone to treat and give us the medication. That is our routine (P)^55^

[T]here are things that I would do my own thing … I know my body better. But I’ll be guided by the doctors (P)^58^

From the other side of the relationship, older patients were viewed as less motivated and potentially lacking the cognitive skills for self-management and those with comorbidities were also thought to lack motivation.^52,54^ Practitioners often expressed a sense of powerlessness to address behaviour change and a hesitancy to approach patients they believed were resistant, and could feel patient’s place responsibility onto them.^22,50,52,57^ However, practitioners also recognised the difficulties patients faced and need to be collaborative and empathetic with patients:

She has got various family problems, money problems, housing problems… In amongst all that … can’t breathe either. It is pulling that big star together of their lifestyle and trying to work out what is going on. (HP)^22^

It’s not pleasant to start exercising, for these patients, it’s not pleasant for anyone who’s unfit to get fit again … it’s much, much worse for them so they need to have that little bit of light at the end of the tunnel, something that they’re aiming for [goals]. (HP)^53^

### Self-management developing over time

Engaging in self-management activities appeared to go hand-in-hand with the length of time living with the condition. Gysels and Higginson^58^ found that:

Over time, patients developed an understanding of their symptoms. Previous experiences put recurring sensations into perspective and constant observation of one’s physical changes, attention to influences from outside and reactions to self-imposed adjustments or treatments, made some people experts in what happened to their body^58^

This notion of patients becoming experts over time was echoed across other papers and linked with subthemes of trial and error, adaption, and normalisation.^33–38,40,41,44^ Trial and error was a process whereby patients came to recognise what they could no longer do and what they were still capable of doing, and in the course of this process adapted their behaviour to accommodate the condition. This materialised in active self-management behaviours such as allowing more time for activities of daily living and hobbies, reducing or changing the kinds of activities engaged in, planning ahead, and also making changes to the home environment.^31,33–38,40,41,44^ While practitioners and the healthcare system were reported to have a role in developing management strategies, patients themselves often undertook this process of adaption without the aid of self-management plans, even when their knowledge of COPD was limited.^33,37^ This revealed an active agency within the population enabling patients to take some control over their condition. By using a “personal filter”^37^ in interpreting advice and adapting to the condition, COPD could become normalised into patient’s lives.

Agency appeared to positively and negatively influence medication use, non-adherence and risk taking behaviour; sometimes in contradiction of medical advice.^33,34,37,42^ For some COPD patients the process of reducing social interaction and slowing down was accepted as an inevitable aspect of the aging process which could help to normalise COPD.^33,38^

Finally, learning to self-manage was often linked to “critical” moments, such as hospitalisation and acute exacerbation episodes that made patients more aware of the consequences of COPD and how their behaviour could impact upon it (e.g., smoking). Critical events offered learning opportunities due to interactions with different professionals and thus information from different sources.^37^

### Social factors

COPD could impact negatively on social interactions due to reduced function and mobility, embarrassment from symptoms (e.g., phlegm, cough) and fear of breathlessness.^39,40,42^ Such limiting of social interaction could lower mood and impact on motivation to engage in self-management activities.^29,30,33,37,39^

I sit on my own and bore myself to death, so I can just as well smoke myself to death (P)^30^

Family members played a key role in emotionally supporting people with COPD to adapt to the condition and engage in self-management behaviours.^29,33,37,40,41,43,47,59^ However, the need to rely on family members could result in ambivalence due to guilt and frustration caused by dependence and changes in family roles;^10,39^ exacerbated by the “invisible” nature of the disease:

Because I don’t look as though I’m ill, the upsetting thing is that people don’t believe you, they think you’re putting it on, sometimes even friends and family. (P)^33^

This frustration could go both ways:

… I’ve been so angry, because he doesn’t ask [question] … he should take a bit of responsibility … for his own life and health (C)^29^

It’s not just affecting him, it’s affecting my sleep … marriage …children… work (C)^59^

In addition, symptoms could reduce sex and intimacy.^42,55^ This could cause relationship tension:

I was a good husband, but for me to make love to my wife was just, certain parts just couldn’t. … because I just get to where I would (deep breath) like I was going to die and she’d just freak out and then she’d just push me away. (P)^42^

Social comparison to others with COPD, or other peers, could have a positive or negative impact on perception of self and the disease.^37,41^ Interacting with others with COPD, particularly through support groups and pulmonary rehabilitation, offered learning opportunities, a sense of validation of lived experience, and an opportunity to make new friendships.^34,37,40,41^

Sheridan et al.,^43^ a New Zealand-based study with a subsample of Pacific Islanders, highlighted that religion or faith could offer a form of support, rationalisation, and acceptance of the condition:

know that it is important for us to pray and keep trying and not just give up, and we are told to wait till death comes. Keep calling our God for his help and wait for his call (P)^43^

### Emotional and psychological factors

Anxiety, panic, and fear were commonly reported by patients, and associated with experiencing breathlessness, hospitalisation, as well as fearing a worsening of symptoms and death.^30,34–38,40–46,59^ This was something recognised by family/carers.^59^ People with COPD faced considerable loss of functional capacity in their lives resulting in frustration, depression, low mood, and worthlessness. McCabe et al. mentioned that even those who did not state that they were depressed commented on “feeling fed up”, “worthless”, “thinking of euthanasia”, “disappointed”, “browned off”, “being vexed”, “having a lack of motivation”, and little “joyfulness”.^40^ Such feelings could hinder motivation:

… you can exercise all you want, but if your heart is aching, you’re feeling depressed, you’ll give up. (P)^41^

However, coming to terms emotionally was deemed important. Humour and determination were both viewed as coping mechanisms:

But you have to make jokes too, don’t you! (P)^34^

I will not let it defeat me … I want to be able to … keep my quality of life going (P)^46^

The role of smoking in causing lung damage could lead to feelings of guilt, self-blame, and shame. These feelings, plus knowledge of the progressive, incurable nature of the disease, could produce a sense of nihilism or helplessness which adversely impacted upon motivation for self-care and adherence to treatment.^39–44^

The emotional issues such as guilt, low mood, and ambivalence were recognised by practitioners.^51,52,55^ Limited consultation times, and the patients’ willingness to disclose and discuss these issues made such support difficult, as exemplified by the excerpts below:

… the emotional part sometimes are not really expressed, because, sometimes we don’t have time … I must say that, actually, they are not coping… but they don’t know how to ask for help. (HP)^55^

### Loss

Living with COPD, learning to manage it and accommodate it, was greatly associated with a sense of loss and slowing down:

There’s a lot of times I can’t even make a cup of coffee (P)^59^

Participants reported loss of function, engagement in activities of daily living and hobbies, social ties, independence, family role, and employment.^31,33–46,55,56,58^ Things that could still be engaged in, needed to be done so in a considered and planned manner:

Even squeezing the toothpaste tube, I have to do it deliberately, not like in the old days when I did it. Boop! Finished! (P)^36^

Furthermore, the condition could be all encompassing, defining the lives of those living with it:

it drains you, it absolutely destroys you (P)^43^

… a living death … it’s just a slow death (P)^59^

it eats up your life (P)^41^

Patients could compensate for losses, substituting previous active hobbies with sedentary ones;^40,41^ however, this could be tinged with disappointment.^41^

As far as my activities are concerned uhhh, I can’t do anything that I appreciate being able to do before I had this disease … all that I used to love to do, can’t do it now so it’s boring, really boring (P)^41^

## Discussion

This review sought to achieve an understanding of barriers and facilitators to COPD self-management from the perspectives of patients and practitioners. Findings suggested that people with COPD are with faced multiple, linked factors which impacted on their ability to engage in self-management, which extended beyond symptom management. For example, while behaviours such as planning ahead and limiting activities could be positive for managing symptoms, such behaviours could also reduce participation and social interaction, resulting in negative emotions and impeded motivation for self-care. Whether something is a barrier or facilitator appeared to be context bound. For instance, families could provide vital emotional and practical support, yet this could instil a sense of guilt or even frustration if support was inadequate. This reflects findings reported elsewhere^7,26,61^ which indicate that factors influencing chronic disease self-management are on a continuum, and can interact, as opposed to being clearly categorised as barriers or facilitators.

Patient’s knowledge and understanding of COPD was often reported as limited. COPD diagnosis and poorer outcomes have been associated with lower socioeconomic status and lower educational attainment.^11–13,62–64^ Low health literacy has been associated with higher disease severity, increased helplessness, poor HRQoL, and greater use of emergency healthcare utilisation in COPD patients.^65^ In addition, health literacy influences health beliefs: Kale et al. found that COPD patients with low health literacy were less likely to believe that COPD is incurable, increasingly likely to express concerns regarding possible negative consequences of medications, and to be concerned about their illness and its effects on their emotions.^66^ Promoting enhanced knowledge within the COPD patient population is a highly complex issue which could be compounded by such social patterning. Gaging patients’ health literacy status could be a vital aspect of self-management support. Nevertheless, some patients are capable of initiating their own management strategies, even when knowledge and understanding is limited. This is, in part, due to developing an understanding of their condition over time. As such practitioners should aim for ongoing engagement with patients accounting for, and harnessing patients’ own illness perceptions and self-learned self-management strategies.^33^ As Effing and colleagues have reported, COPD self-management interventions should be iterative, patient-centred interactions, that account for literary and health literacy focusing on:

1) identifying needs, health beliefs and enhancing intrinsic motivations; 2) eliciting personalised goals; 3) formulating appropriate strategies (e.g. exacerbation management) to achieve these goals; and if required 4) evaluating and re-adjusting strategies.^8^

Achieving this requires comprehensive training for practitioners across the healthcare system, which our findings suggest, is currently lacking. Some practitioners had a narrow view of self-management, focused on symptom management, and some lacked the awareness, skills and/or confidence to engage with COPD patients beyond established practice and patients could feel that practitioners lead consultations. Such issues are acknowledged in the wider literature. Even when practitioners do recognise wider psychosocial needs, they often lack consultation time to effectively discuss patient concerns, a problem that extra education and training alone would not fully address.^61^ Where practitioners recognise patient autonomy they can struggle to reconcile this autonomy with achieving positive medical outcomes and active patient involvement, and can prefer to maintain traditional patient-practitioner boundaries.^67^ Thus there should be a greater focus on interventions that encourage and enable practitioners to cultivate effective ways of engaging with patients, such as shared decision-making.^68^

The emotional and psychological burden of chronic illness is well established.^4–7,23,69–73^ Depression and anxiety are common among people with COPD as are feelings of guilt and stigma due to the perceived self-inflicted nature, and visible aspects of COPD.^74–76^ Family members can help to quell emotional factors; however, some patients may be reluctant to discuss sensitive issues with already burdened carers. Thus a supportive family should not necessarily be viewed as a proxy for emotional support by practitioners; emotional and mental health needs should always be acknowledged and addressed in consultations. In some cases, there may be a need for family-centred self-management interventions to both support patients and help avert feelings of burden, stress, and burnout.^77–79^

COPD patients experience a range of losses and isolation and the condition can seem to consume their existence. Gullick and Stainton theorised that living with breathlessness leads to a “shrinking lifeword”, where:

“loss of taken-for-granted breathing increasingly limited the person’s self-care abilities, social activities, hobbies and mobility” and “diminishes the predictability and automatic nature of [their] bodies and [their] perceived effectiveness as a person”^80^

This speaks to the wider literature on the lived experience of chronic conditions which highlights the potential disruption and change caused to biography and identity.^69–73,81^ Hence there is a need to recognise individuals’ identity, history, and lifeworld within the sphere of treatment.^72,80^ Indeed, self-management interventions incorporating behaviour change techniques targeting mental health have been shown to be more effective than those that focus on symptom management alone.^17,20^

There was heterogeneity across the self-management definitions identified in this review. The over-focus on knowledge and skills, and under-focus on emotional factors and HRQoL, is problematic. It is well established that chronic conditions impact upon HRQoL and can present an emotional burden. Addressing these issues must go hand-in-hand with bio-medical disease management (e.g., symptom monitoring, managing medications).^8^ The promotion of holistic and comprehensive descriptions of self-management could help to guide research, policy, and practice (see Effing et al.^8^).

### Limitations

This review used robust systematic review methods (e.g., search, screening, extraction, appraisal process) and identified a rich body of relatively high-quality research. Exploring patient, carer, and practitioner accounts also provided a rounded understanding of self-management from multiple perspectives, with clear practice and policy implications.

This qualitative review was limited by the original reporting of data by paper authors and the selection of quotes they used in papers. The female voice was under represented in this literature, and there was little representation of the working age population. Ethnic diversity appeared to be limited; however, this was unclear due to the lack of specific reporting within the papers.

By focusing on self-management within the search, as opposed to the experiences of living with and managing COPD more broadly, this review could have missed relevant sociological and social science literature. However, we searched five key databases, and specifically sought qualitative, multidisciplinary work.

### Conclusion and implications

Living with COPD is a complex, individual experience and thus the ability and capability of people with COPD to engage in successful self-management is dependent on their own personal life context, attitudes, beliefs, emotional responses, socio-cultural resources, and time living with the condition. Primary care and community-based practitioners are well placed to provide self-management support that is tailored and personalised to patients with whom they often have well-established relationships.^82–84^ Support should be collaborative, addressing how patients conceptualise their condition, the kinds of adaptions they have made to accommodate their condition in order to harness the patient’s pre-existing capabilities and motivation, and address wider psychosocial issues.

## Methods

The review protocol was pre-registered with PROSPERO (CRD42016040169).

### Literature identification

The search strategy (Supplementary Table 1) was designed in collaboration with an experienced information specialist [F.B.], who used thesaurus headings and keywords relating to the COPD and self-management, and translated as appropriate for each database. A multidisciplinary range of databases, most likely to retrieve qualitative research papers, were used. The following databases were searched up to October 2017: MEDLINE (Ovid); ASSIA (ProQuest); CINAHL (EBSCO); PsycInfo (Ovid); ISI Web of Knowledge. The search was restricted to peer-reviewed articles published in English.

### Inclusion and exclusion criteria

To qualify for inclusion, studies needed to focus on COPD self-management (including where comorbidities were present), from the perspective of patients, their family or carers, and/or practitioners. Studies needed to use qualitative data collection (e.g., focus groups, interviews), analysis, and reporting via narrative data.

Papers were excluded if: (i) there were insufficient or no narrative data to support findings; (ii) there was a focus on intervention outcomes (this review did not aim to compare interventions); (iii) the focus was on behaviour change (e.g., smoking cessation, management of exacerbation) without exploring self-management more broadly; (iv) if participants were experiencing end of life, or palliative care; (v) if the population contained multi-morbidity without a specific focus on COPD.

### Screening

Articles were screened on title and abstract by one researcher [S.R.] and 20% were independently screened by a second researcher [O.O.]. Articles which met inclusion criteria, or could not be excluded based on the title and abstract alone, were retrieved and checked. Additional researchers [J.N., E.K.] assisted in settling discrepancies.

### Quality assessment

Quality appraisal of qualitative research is a contested issue, partly due to variation in qualitative methodologies and what is considered “good” work within differing approaches.^85,86^ Despite this, assessing quality can prevent unreliable findings from having undue influence on the results of a review.^87^ Thus all full-text articles were appraised.

Quality appraisal criteria were adapted from a pre-existing tool focusing on credibility, depth and richness findings, relevance, and whether or not findings are transferable to other settings.^88^ These criteria were assessed on the basis of reporting being judged to be “Very good”, “Good”, or “Limited” (Supplementary Table 2).

### Data extraction and synthesis

Data were extracted by two researchers [S.R., O.O.] (Supplementary Table 3). The wider team supported extraction of factors viewed as barriers or facilitators to self-management. Themes presented by paper authors and data excerpts were compared and contrasted across the body of work, and then grouped and translated into superordinate (higher level) themes via an iterative process, representing cumulative findings. Data excerpts were selected to exemplify our over-arching themes.

## Electronic supplementary material

Supplementary Table 1

Supplementary Table 2

Supplementary Table 3
