# Supplementary material for: Qualitative systematic review of barriers and facilitators to self-management of chronic obstructive pulmonary disease: views of patients and healthcare professionals
Source: NPJ Prim Care Respir Med. 2018 Jan 17;28:2. doi: 10.1038/s41533-017-0069-z (PMC5772437; doi:10.1038/s41533-017-0069-z)
Supplement: Supplementary file 1 — Supplementary Table 1 [file 41533_2017_69_MOESM1_ESM.docx]

**Supplementary Table 1 Search Strategy**

| **Date of searches: October 2016** | | | |
| --- | --- | --- | --- |
| **Key** | | | |
| exp | | | Explode thesaurus heading/subject heading |
| / or MH | | | Denotes the term is a thesaurus heading |
| $ | | | Truncation; retrieves any ending |
| adjx or Nx | | | Proximity; dictates words must be within x words of each other in the record |
| .tw | | | Words must appear in title or abstract field |
| .ti, .ab, .id | | | Words must appear in title, abstract, keyword (PsycINFO only) field |
| Or/1-5 | | | 1 or 2 or 3 or 4 or 5 |
| **Database(s): Ovid MEDLINE(R) In-Process & Other Non-Indexed Citations and Ovid MEDLINE(R)**  **Search strategy:** | | | |
| # | | Searches | |
| 1 | | exp Pulmonary Disease, Chronic Obstructive/ | |
| 2 | | emphysema$.tw. | |
| 3 | | (chronic$ adj3 bronchiti$).tw. | |
| 4 | | (obstruct$ adj3 (pulmonary or lung$ or bronch$ or respirat$)).tw. | |
| 5 | | (COPD or COAD or COBD or AECB).tw. | |
| 6 | | or/1-5 | |
| 7 | | exp Self Care/ | |
| 8 | | (self-manag$ or self manag$ or self-car$ or self car$ or self-administ$ or self administ$).tw. | |
| 9 | | (patient$ adj3 (focus$ or participat$ or centr$ or center$ or empower$ or support$ or collaborat$ or co-operat$ or cooperat$)).tw. | |
| 10 | | or/7-9 | |
| 11 | | 6 and 10 | |
| 12 | | interviews as topic/ or focus groups/ or narration/ or qualitative research/ | |
| 13 | | ((("semi-structured" or semistructured or unstructured or informal or "in-depth" or indepth or "face-to-face" or structured or guide) adj3 (interview$ or discussion$ or questionnaire$)) or (focus group$ or qualitative or ethnograph$ or fieldwork or "field work" or "key informant")).ti,ab. | |
| 14 | | 12 or 13 | |
| 15 | | 11 and 14 | |
| **Database: PsycINFO (OVID)**  **Search Strategy:** | | | |
| # | | Searches | |
| 1 | | exp Chronic Obstructive Pulmonary Disease/ | |
| 2 | | emphysema$.ti,ab,id. | |
| 3 | | (chronic$ adj3 bronchiti$).ti,ab,id. | |
| 4 | | (obstruct$ adj3 (pulmonary or lung$ or bronch$ or respirat$)).ti,ab,id. | |
| 5 | | (COPD or COAD or COBD or AECB).ti,ab,id. | |
| 6 | | or/1-5 | |
| 7 | | exp Self Management/ | |
| 8 | | (self-manag$ or self manag$ or self-car$ or self car$ or self-administ$ or self administ$).ti,ab,id. | |
| 9 | | (patient$ adj3 (focus$ or participat$ or centr$ or center$ or empower$ or support$ or collaborat$ or co-operat$ or cooperat$)).ti,ab,id. | |
| 10 | | or/7-9 | |
| 11 | | 6 and 10 | |
| 12 | | (exp qualitative research/ or exp interviews/ or exp group discussion/ or qualitative study.md.) not "Literature Review".md. | |
| 13 | | ((("semi-structured" or semistructured or unstructured or informal or "in-depth" or indepth or "face-to-face" or structured or guide or guides) adj3 (interview* or discussion* or questionnaire*)) or (focus group* or qualitative or ethnograph* or fieldwork or "field work" or "key informant")).ti,ab,id. | |
| 14 | | 12 or 13 | |
| 15 | | 11 and 14 | |
| **Database: CINAHL (EBSCO)**  **Search Strategy:** | | | |
| # | | Searches | |
| 1 | | (MH "Pulmonary Disease, Chronic Obstructive+") | |
| 2 | | TI emphysema* OR AB emphysema* | |
| 3 | | TI chronic* N3 bronchiti* OR AB chronic* N3 bronchiti* | |
| 4 | | TI ( obstruct* N3 (pulmonary or lung* or bronch* or respirat*) ) OR AB ( obstruct* N3 (pulmonary or lung* or bronch* or respirat*) ) | |
| 5 | | TI ( COPD or COAD or COBD or AECB ) OR AB ( COPD or COAD or COBD or AECB ) | |
| 6 | | S1 OR S2 OR S3 OR S4 OR S5 | |
| 7 | | (MH "Self Care+") | |
| 8 | | TI ( self-manag* or self manag* or self-car* or self car* or self-administ* or self administ* ) OR AB ( self-manag* or self manag* or self-car* or self car* or self-administ* or self administ* ) | |
| 9 | | TI ( patient* N3 (focus* or participat* or centr* or center* or empower* or support* or collaborat* or co-operat* or cooperat*) ) OR AB ( patient* N3 (focus* or participat* or centr* or center* or empower* or support* or collaborat* or co-operat* or cooperat*) ) | |
| 10 | | S7 OR S8 OR S9 | |
| 11 | | S6 AND S10 | |
| 12 | | (MH "Qualitative Studies+") | |
| 13 | | (MH "Attitude+") | |
| 14 | | (MH "Interviews+") | |
| 15 | | TI ( ("semi-structured" or semistructured or unstructured or informal or "in-depth" or indepth or "face-to-face" or structured or guide) N3 (interview* or discussion* or questionnaire*) ) OR AB ( ("semi-structured" or semistructured or unstructured or informal or "in-depth" or indepth or "face-to-face" or structured or guide) N3 (interview* or discussion* or questionnaire*) ) | |
| 16 | | S12 OR S13 OR S14 OR S15 | |
| 17 | | S11 AND S16 | |
| **Database: ASSIA (ProQuest)**  **Search Strategy:** | | | |
| (SU.EXACT.EXPLODE("Chronic obstructive pulmonary disease") OR (TI(obstruct* NEAR/3 (pulmonary OR lung* OR bronch* OR respirat*)) OR AB(obstruct* NEAR/3 (pulmonary OR lung* OR bronch* OR respirat*))) OR (TI(chronic NEAR/3 bronchiti*) OR AB(chronic NEAR/3 bronchiti*)) OR (TI(emphysema*) OR AB(emphysema*)) OR (TI(COPD OR COAD OR COBD OR AECB) OR AB(COPD OR COAD OR COBD OR AECB))) | | | |
| AND | | | |
| (SU.EXACT.EXPLODE("Selfcare") OR (TI(self-manag* OR self manag* OR self-car* OR self car* OR self-administ* OR self administ*) OR AB(self-manag* OR self manag* OR self-car* OR self car* OR self-administ* OR self administ*)) OR (TI(patient* NEAR/3 (focus* OR participat* OR centr* OR center* OR empower* OR support* OR collaborat* OR co-operat* OR cooperat*)) OR AB(patient* NEAR/3 (focus* OR participat* OR centr* OR center* OR empower* OR support* OR collaborat* OR co-operat* OR cooperat*)))) | | | |
| **Database: Web of Science**  **Search Strategy:** | | | |
|  | Searches | | |
| 1 | TI=COPD OR TI=emphysema* OR TI=(chronic* NEAR bronchitis*) | | |
| 2 | TI=(obstruct* NEAR (pulmonary or lung* or bronch* or respirat*)) | | |
| 3 | TI=(COPD or COAD or COBD or AECB) | | |
| 4 | #3 OR #2 OR #1 | | |
| 5 | TS=(self-manag* or self manag* or self-car* or self car* or self-administ* or self administ*) | | |
| 6 | TS=(patient* NEAR (focus* or participat* or centr* or center* or empower* or support* or collaborat* or co-operat* or cooperat*)) | | |
| 7 | #6 OR #5 | | |
| 8 | TS=(focus group* or qualitative or ethnograph* or fieldwork or "field work" or "key informant") | | |
| 9 | TS=(interview* or discussion* or questionnaire*) | | |
| 10 | #9 OR #8 | | |
| 11 | #10 AND #7 AND #4 | | |
