# Supplementary material for: Qualitative systematic review of barriers and facilitators to self-management of chronic obstructive pulmonary disease: views of patients and healthcare professionals
Source: NPJ Prim Care Respir Med. 2018 Jan 17;28:2. doi: 10.1038/s41533-017-0069-z (PMC5772437; doi:10.1038/s41533-017-0069-z)
Supplement: Supplementary file 2 — Supplementary Table 2 [file 41533_2017_69_MOESM2_ESM.docx]

**Supplementary Table 2 Quality Appraisal**

| **Domain** | **Considerations** |
| --- | --- |
| Aims and scope | Is the research question clearly defined?  Are the aims/objectives clearly defined?  Is the scope of the research detailed? |
| Ethical dimensions | Ethical committee approval granted?  Documentation of how autonomy, consent, confidentiality, anonymity were managed? |
| Study design | Is the design apparent, and consistent with research intent?  Is the data collection strategy apparent and appropriate?  Who collected the data?  Procedures?  Rationale for the study design discussed? |
| Rigour in research conduct | Rationale for the study design justified?  Is a sampling strategy well defined and justified?  Is the method of data collection clearly described?  Analysis procedure transparent? |
| Analysis procedure | Is the method of analysis clearly described?  Evidence of engagement with other researchers to reduce researcher bias?  Discussion of how coding systems/conceptual frameworks evolved?  Evidence provided that data reached saturation or discussion/rationale if not? |
| Depth, detail, and richness of findings  E.g. “thin vs. thick description” | Thin: lacks detail and/or consideration of context, little explanatory insights  Thick: consideration of context, detail, greater potential for explanation and generalization  Do the findings privilege subjective experience and meanings? |
| Credibility of interpretation | Are the claims made supported by sufficient evidence?  Appropriate use of data [participant quotes etc.] in terms of relevance and number of incidences?  Is it clear data whether multiple participants are used to frame findings or whether one voice dominates the presentation of findings? |
| Relevance and transferability of findings | Detailed description of the context of the study to allow assessment of applicability to other settings?  Is the description provided detailed enough to allow the researcher or reader to interpret the meaning and context of what of being researched?  Discussion of limits to wider inference? |
| Contribution to knowledge | Clear discussion of how the research findings contribute to knowledge within focus area (e.g self-management and COPD)  New areas of investigation identified? |
