# Supplementary material for: Qualitative systematic review of barriers and facilitators to self-management of chronic obstructive pulmonary disease: views of patients and healthcare professionals
Source: NPJ Prim Care Respir Med. 2018 Jan 17;28:2. doi: 10.1038/s41533-017-0069-z (PMC5772437; doi:10.1038/s41533-017-0069-z)
Supplement: Supplementary file 3 — Supplementary Table 3 [file 41533_2017_69_MOESM3_ESM.docx]

**Supplementary Table 3 Data Extraction**

| **Categories** | **Data extracted** |
| --- | --- |
| **Objective Categories** |  |
| **Article overview** | Citation |
|  | Country |
|  | Aim(s)/Objective(s)/Research question(s) |
|  | Background/grounding |
|  | Definition/description of 'self-management' |
| **Method(s)** | Study design and methodological approach |
|  | Type of study |
|  | Recruitment context and sampling strategy |
|  | Inclusion and exclusion criteria |
|  | Date of data collection |
|  | Data collection |
|  | Interview topic guide |
|  | Data analysis |
|  | Efforts to establish validity and reliability |
|  | Ethics approval |
| **Results / Findings** | Number and type of participants (e.g. patients, family/carers, HCPs, others) |
|  | Participants' characteristics |
|  | Author presented themes (theme name and description) |
|  | Data Extracts (Quotes, others) |
|  | Author explanation/discussion of findings |
|  | Recommendations (practice, policy, research) |
|  | Author identified bias/study limitations Reviewer identified bias/study limitations |
| **Subjective Categories** |  |
| **Reviewer identified themes related to review focus (e.g. factors relating to self-management of COPD)** | Themes relating to barriers to self-management  Themes relating to facilitators to self-management |
